# Perspectives on Energy Transport at the Micro/Nanoscale

**DOI:** 10.3390/nano13111746

**Published:** 2023-05-26

**Authors:** Xinwei Wang

**Affiliations:** Department of Mechanical Engineering, Iowa State University, Ames, IA 50011, USA; xwang3@iastate.edu; Tel.: +1-515-294-8023

Over the last two decades, with the fast development of micro/nanomaterials, including micro/nanoscale and micro/nanostructured materials, significant attention has been attracted to study the energy transport in them [1,2,3]. This energy transport can be sustained by electrons and phonons, whose transport is strongly affected by micro/nanoscale structure scattering. Numerous computer models based on first-principle, molecular dynamics, and lattice Boltzmann transport have been reported [4,5,6]. Also tremendous efforts have been reported on development of new technologies to characterize the thermophysical properties at the micro/nanoscale [7,8]. Examples of such properties include thermal conductivity, thermal diffusivity, and specific heat [9,10,11].

Thermophysical characterization at the micro/nanoscale is extremely challenging due to the very small size of the sample under study. It is a non-trivial job to apply a very well defined heat flux along the sample and characterize the temperature drop over it. To overcome this challenge, new transient techniques have been developed by applying transient Joule or photon heating, and track the material’s thermal response using electrical or optical methods. Examples include the transient electro-thermal (TET), transient photo-electro-thermal (TPET), pulsed laser-assisted thermal relaxation (PLTR), time domain differential Raman (TD-Raman), frequency-resolved Raman (FR-Raman), and energy transport state-resolved Raman (ET-Raman) techniques [7,8,12,13,14,15,16]. These techniques provide quick and high-level measurement of thermal diffusivity/conductivity of 1D and 2D materials down to atomic-level thickness [17].

This special issue includes some recent work on micro/nanoscale energy transport, including research progress reports and reviews. These reviews are in great technical detail since the authors have tremendous experience and expertise in the topics under review. In the paper by Lin et al. [18], the TET technique was reviewed thoroughly, especially about the differential TET concept. The TET technique measures 1D material’s voltage/resistance change under step-current heating and can be used to measure thermal diffusivity, conductivity, and specific heat. The differential concept is especially useful in measuring materials of nm-thickness which is too thin to suspend between two electrodes. The energy transport in 2D materials features very unique characteristics due to the extreme phonon confinement and scattering. The review by Kalantari and Zhang [19] provides excellent coverage, discussions, and perspectives on computer modeling and experimental characterization. The works by Dai and Wang [20] and Liu et al. [21] are great additions to the review by Mohammad and Xian, with focus on detailed experimental development. The work by Zhou et al. [22] provides sound technical details on the photothermal technique for measuring the thermal conductivity and interface thermal resistance of coatings. This work covers the fundamental physical principles, mathematical solutions for data processing, and typical examples of measurement.

Of the research progress reports covered by this Special Issue, the work by Xu et al. provides inspiring measurement technique and knowledge about the dynamic thermal diffusivity evolution during fast heating of corn leaves and ultra-high-molecular-weight polyethylene (UHMW-PE) micro-fibers [23]. This indeed significantly extends the capability of the TET technique and provides unprecedented knowledge about thermal diffusivity evolution during fast heating. The work by Liu et al. presents pioneering efforts in studying the thermal conductivity variation of giant-scale graphene depending on temperature [24]. This work is very challenging since the thermal expansion mismatch between graphene and the poly(methyl methacrylate) (PMMA) substrate could easily break the graphene layer when temperature goes down. The experimental work by Wang et al. on the bolometric response of MoS_2_ nanoflowers and multi-walled carbon nanotube composite [25], and work on the effect of current annealing on thermal conductivity of carbon nanotubes by Lin et al. [26] present welcome efforts on studying the structural and temperature effects on energy transport. Nanoscale energy transport has tremendous applications in materials synthesis. The work by Deng et al. [27] reports the microstructure and superior corrosion resistance of NiTi-based intermetallic coatings. Such coating synthesis uses laser melting deposition, which is a strong energy transport-controlled process. The work by Nunes et al. reported detailed study of electrochemical behavior related to charge transport in double-layer capacitors and pseudocapacitors [28]. An understanding of the physics of such transport phenomenon is critical to the design and optimization of capacitor performance.

Energy transport at the micro/nanoscale is still a very active research area, and current research is very diverse, including study of detailed phonon dynamics, structure, and behaviors, material design to either enhance or suppress energy transport, and new technology development to overcome challenges in characterizing special materials [29,30].
